# Effects of Vitamin D Deficiency on Incidence Risk of Gestational Diabetes Mellitus: A Systematic Review and Meta-analysis

**DOI:** 10.3389/fendo.2018.00007

**Published:** 2018-02-01

**Authors:** Mansour Amraei, Safoura Mohamadpour, Kourosh Sayehmiri, Seyedeh Fatemeh Mousavi, Ehsan Shirzadpour, Ardeshir Moayeri

**Affiliations:** ^1^Biotechnology and Medicinal Plants Research Center, Ilam University of Medical Sciences, Ilam, Iran; ^2^Faculty of Medicine, Department of Physiology, Ilam University of Medical Sciences, Ilam, Iran; ^3^Psychosocial Injuries Research Center, Ilam University of Medical Sciences, Ilam, Iran; ^4^Faculty of Medicine, Department of Anatomy, Ilam University of Medical Sciences, Ilam, Iran

**Keywords:** gestational diabetes mellitus, vitamin D deficiency, pregnancy, systematic review, meta-analysis

## Abstract

**Introduction:**

Proper nutrition is important for overall health, and it reduces healthcare costs associated with malnutrition. Many studies have investigated vitamin D deficiency and its role in gestational diabetes and controversial data have reported. A comprehensive consideration of articles in this field provides the possibility of a general study of this relationship. This meta-analysis is an evaluation of the relationship between vitamin D deficiency and gestational diabetes.

**Material and methods:**

Different databases (such as PubMed, Science Information Institute, EmBase, Scopus, and the Cochrane Library) were searched for studies and eligible English articles published before February 2017 that have reported the risk of gestational diabetes in relation to vitamin D deficiency. This relationship was measured using odds ratios (ORs) with a confidence interval (CI) of 95%. The influence of each study was measured through sensitivity analysis. Funnel plots, Egger regression tests, and the Begg–Mazumdar correlation test were used to determine bias or publication bias. STATA (version 11.2) was used for all analyses.

**Results:**

Twenty-six studies were selected as eligible for this research and included in the final analysis. In general, vitamin D deficiency among mothers may be related to an increased risk of gestational diabetes (OR = 1.18; 95% CI, 1.01–1.35; *p* < 0.001). The serum level of 25(OH)D is less meaningful in people with gestational diabetes than in those who have normal glucose tolerance. Subgroup analysis showed that the results concerning this association may vary with study design but do not change with country of origin.

**Conclusion:**

Some evidence has shown that vitamin D deficiency may increase the risk of gestational diabetes.

## Introduction

Gestational diabetes mellitus (GDM), a frequent disorder in pregnancy, is defined as any degree of glucose intolerance that first develops or is first recognized during pregnancy (1, 2). The incidence of GDM is rapidly increasing worldwide, and it affects 1–25% of all pregnancies depending on patient demographics, screening strategies, and diagnostic thresholds (2, 3). According to the Hyperglycemia and Adverse Pregnancy Outcome Study Cooperative, GDM increases the number of maternal and fetal complications (4–6).

It has been hypothesized that vitamin D deficiency is a risk factor associated with GDM. The need for vitamin D is higher during specific stages of life, including during the rapid growth of the fetus in the embryonic stage, infancy, the early stages of childhood, puberty, and pregnancy (6). In addition to its classical roles in influencing calcium absorption and bone metabolism, vitamin D has been shown to be associated with nonskeletal conditions (7, 8). Several recent studies have shown the possible effect of vitamin D supplementation in reducing the risk of fetal complications in pregnancies where the mother has vitamin D deficiency (9–11). These studies revealed that vitamin D deficiency is common in pregnancy and significantly increases the risk for preeclampsia, cesarean section, and GDM in later pregnancy (9–13).

Recent evidence suggests that vitamin D receptors are expressed in many different cells, including those that influence the regulation of glucose metabolism such as muscle and pancreatic beta cells (14). Vitamin D has a direct effect on pancreatic beta cells and is a prerequisite for the normal insulin secretion function of the endocrine pancreas (15, 16). Therefore, vitamin D deficiency is related to blood glucose and insulin concentration alterations as well as target tissue sensitivity to insulin (6). Vitamin D replenishment restores insulin secretion and sensitivity in patients with type 2 diabetes and established vitamin D deficiency (17). Therefore, it was hypothesized that GDM might result from pregnancy-induced insulin resistance and the resulting impaired insulin secretion.

The association of vitamin D with the risk of gestational diabetes has long been of interest to researchers, and many studies in a broad variety of populations have been conducted on this topic, yielding different outcomes. Some studies have showed a meaningful relationship between vitamin D status and risk of gestational diabetes whereas others have reported controversial data. With this large body of literature, an overall estimation of its association is important, as is indicated by the breadth and quality of these studies. Recently, several meta-analyses have explored the existing data on the association of serum vitamin D levels with the risk of GDM and have reported that such association indeed exists (18–21). Despite these findings, there is also data to the contrary and our knowledge about the clinical importance and implications of this association is still limited. Further studies have been published since the initial appearance of these meta-analyses, but they have not yet been effectively summarized. To authenticate these studies and as a way of synthesizing their findings, this updated meta-analysis was performed to quantitatively evaluate the association of vitamin D status (deficient vs. normal) with the risk of gestational diabetes.

## Materials and Methods

### Search Strategy

This meta-analysis was done on the basis of the PRISAMA statement about meta-analyses (check list S1) (22). Two researchers performed formal, independent computer searches for related published articles (to February 2017) with existent documents in the Scopus databases, Information Sciences Institute website, PubMed, EmBase, and the Cochrane Library. Keywords searched included vitamin D, 25-hydroxy vitamin D, 1,25-dihydroxycholeclciferol, and 25(OH)D with GDM. The meta-analysis was limited to studies published in English. The search scope was developed using the symbol “*” and an advanced search of words or statements created by Boolean operators was performed. The list of recognized articles was scanned, and the reference lists of all related reviews and main articles were searched manually for more references. To decrease bias, two authors performed the searches independently, and any disagreement between them was debated in a group discussion until a consensus was achieved.

### Inclusion and Exclusion Criteria

For further review, abstracts and titles were screened by two reviewers. Since every screened study was included in this meta-analysis, the researchers attempted to evaluate the relationship between vitamin D and the risk of GDM; determine whether it is accomplished among pregnant women without illness or as a chronic complication; use blood samples for lab tests; compare women with gestational diabetes and women with a normal glucose tolerance (NGT); report an estimation of effect [ratios risk (RR), hazard risk (HR), or odds ratio (OR) and related confidence interval (CI) of 95%] to compare adequate and inadequate vitamin D values. Some studies were out of the realm of the current study, which focused on pregnant women with chronic illness, and thus were excluded. Studies on non-human creatures (i.e., animal studies), those published in languages other than English, those that were meta-analyses or systematic considerations, and those that presented insufficient data or were duplicate publications were also excluded.

### Data Extraction

Data and information on the basis of a standard protocol were excluded after the determination was made for eligible articles. The data collected included the name of first writer, publication year, sample size, age group, present situation, study design, evaluation of the vitamin D status, and the effect estimation with a CI of 95%. In cases which needed more information, the articles’ writers were contacted for supplementary data or further elucidation. Information from articles by two specialists who worked independently was considered, and their findings and results were compared later. Disagreements about study eligibility were resolved by group discussion. The information and data were entered in a standard data extraction form and finally into Microsoft Excel.

### Data Synthesis and Analysis

Reported effect estimation (OR and RR) with a CI of 95% was used as the measuring standard of the relation between vitamin D and the risk of gestational diabetes. The possible estimation of risk was maximized to evaluate the variance between some methods and levels which control studies of potential confounding factors. Statistical heterogeneity was evaluated in studies using *Q* and *I*^2^ Cochran statistics. Wherever the results of studies were heterogeneous, a random effects model was used in the meta-analysis. Subgroup analyses were carried out in search of possible heterogeneous causes. Integrated estimations and the related CI of 95%were evaluated using forest plots as visuals. Bias or publication bias was evaluated as quality using funnel plots, Egger regressions, and the Begg–Mazumdar correlation test.

Values of *p* < 0/05 were considered as valid for heterogeneity tests. For statistical analyses, software R (version 3.2.1) and STATA (version 11.1) software were used. All statistical tests were two sided.

## Results

### Selected Articles

**Figure 1:**
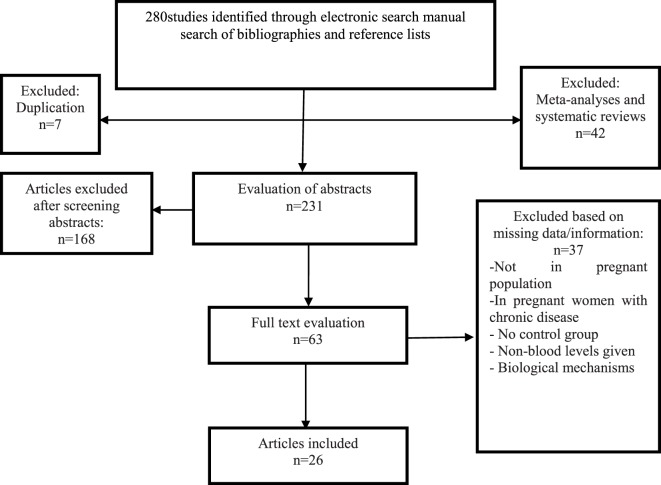
Flowchart of the literature search.

In the initial electronic search, 268 potential articles were identified; a manual search of the bibliographies and reference lists of these articles identified 12 additional articles. Altogether, 280 articles were identified through the literature search. After the initial screening of abstracts and titles, 217 articles were excluded based on the inclusion criteria (49 articles based on unrelated titles and 168 based on unrelated abstracts) and 63 articles remained for full text review. In a secondary screening and after a full-text review, another 37 articles were excluded; 4 studies were not in a population of pregnant women, 8 studies were in pregnant women with chronic diseases or conditions, 6 studies did not obtain blood samples for testing, 3 studies reported on biological mechanisms of vitamin D metabolites, 7 studies had no outcome data, and 6 studies collected insufficient data. Two studies were non-human (animal) studies, and one was not published in English. Twenty-six studies were selected for the final analysis after these exclusions (Figure 1).

### Description of the Studies

The studies used in this meta-analysis were published between 2008 and 2016 and consisted of eight cross-sectional studies (7, 15, 25, 30, 31, 43, 45, 46), six prospective, nested case–control studies (28, 33, 35–37, 44), seven retrospective case–control studies (6, 17, 23, 24, 27, 29, 40), and five prospective cohort studies (16, 26, 32, 38, 47). All 26 articles presented their findings as proportions (Table 1).

**Table 1:** Characteristics and information of studies evaluated in this meta-analysis.

Reference	Country	Study design	Sample size (*n*)		Age at baseline (year)	Current status	Assay method for vitamin D^a^	Mean 25(OH)D nmol/L (SD)		Significant
			GDM	NGT				GDM	NGT	
(43)	Australia	Cross-sectional	81	226	32.6	Second or Third trimester	LC–MS	48.6 (24.9)	55.3 (23.3)	No
(44)	US	Nested Case–control	57	114	33.5	24–28 weeks	ELISA	60.4 (21.22)	75.13 (24.21)	Yes
(15)	Iran	Cross-sectional	52	579	25.6	24–28 weeks	RIA	16.49 (10.44)	22.97 (18.25)	No
(45)	India	Cross-sectional	39	520	23.7	<32 weeks	RIA	49.3 (31.2)	46.4 (30.9)	No
(23)	Iran	Case–control	54	111	27.4	24–28 weeks	ELISA	24.01 (20.62)	32.2 (35.74)	No
(29)	UK	Case–control	90	158	33.5	First trimester	LC–MS	47.2 (26.7)	47.6 (26.7)	No
(24)	UK	Case–control	100	1,000	31.7	11–19 weeks	LC–MS	NR	NR	No
(35)	US	Nested-case–control	60	120	33.7	24–28 weeks	LC–MS	97.0 (29.0)	86.0 (22.0)	No
(36)	Canada	Nested-case–control	116	218	34.3	15–18 weeks	CLIA	56.3 (19.4)	62 (21.6)	No
(37)	China	Nested-case–control	200	200	32	26–28 weeks	ELISA	22.4 (10.7)	25.9 (12.3)	Yes
(46)	Spain	Cross-sectional	36	466	NR	11–14 weeks	ECLIA	NR	NR	NR
(30)	Spain	Cross-sectional	49	266	NR	24–28 weeks	CLIA	NR	NR	No
(31)	US	Cross-sectional	68	1,264	32	26–28 weeks	CLIA	NR	NR	No
(25)	Turkey	Cross-sectional	234	168	30.8	24–28 weeks	ECLIA	30.8 (16.3)	36.0 (16.2)	Yes
(26)	Qatari	Cohort	260	1,613		>24 weeks	RIA	44.19 (20.01)	NR	Yes
(27)	Turkey	Case-control	44	78	26.4	24–32 weeks	CLIA	48.67 (23.21)	57.16 (24.96)	Yes
(28)	Australia	Nested case–control	376	3,714	33.2	First trimester	AIAS	56.9 (26.9)	52.1 (22.1)	No
(7)	Canada	Cross-sectional	54	601	28.4	6–13 weeks	LC–MS	57.5 (17.2)	63.5 (18.9)	No
(32)	Korea	Cohort	23	500	33.7	24–28 weeks	ECLIA	49.4 (19.4)	48 (24.8)	No
(38)	China	Cohort	2,960	100	29.7	16–20 weeks	ECLIA	NR	NR	No
(47)	Spain	Cohort	93	2,289	32	13.5 weeks	HPLC	28.42 (4.39)	28.41 (0.96)	No
(16)	Canada	Cohort	142	125	34.4	NR	ECLIA	NR	NR	No
(33)	US	Nested-case–control	135	517	33.5	18–22 weeks	LC–MS	59.7 (23.5)	66.6 (22)	No
(40)	Czech	Case–control	47	29	33	24–30 weeks	EIASA	28.5 (13)	31.7 (16)	No
(6)	Iran	Case–control	43	44	31.28	First trimester	Liebermann–Burchard method	33.54 (18.88)	43.23 (37.02)	Yes
(17)	India	Case–control	51	19	26.5	<28 weeks	RIA	24.7 (17.6)	45.8 (28)	Yes

*Significant: significant difference in serum 25(OH)D between NGT and GDM*.

*^a^Assay method of 25(OH)D*.

*NR, not reported; LC–MS, liquid chromatography-tandem mass spectrometry; ELISA, enzyme-linked immunosorbent assay; RIA, radioimmunoassay; CLIA, chemiluminescence immunoassay; ECLIA, ElectroChemiLuminescence immunoassay; AIAS, automated immunoassay system; HPLC, high-performance liquid chromatography*.

### Main Analysis

**Figure 2:**
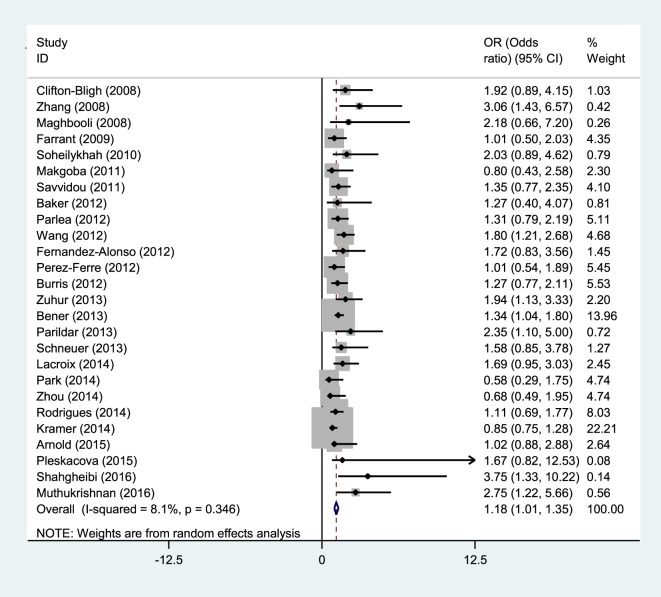
Relationship between insufficient vitamin D and risk of GDM.

Studies reporting OR were collected to determine the association between vitamin D deficiency and gestational diabetes. Seven studies showed a meaningful relationship between vitamin D status and risk of gestational diabetes, and 19 other cases showed no significant relationship. Thus, the present meta-analysis used a random effects model to show that vitamin D deficiency is related to an increased risk of GDM (OR = 1.18; 95% CI, 1.01–1.35; *p* < 0.001) (Figure 2). The reviewed studies showed limited evidence of heterogeneity (*I*^2^ = 8.1%; *p* = 0.346).

### Sensitivity and Subgroup Analyses

**Figure 3:**
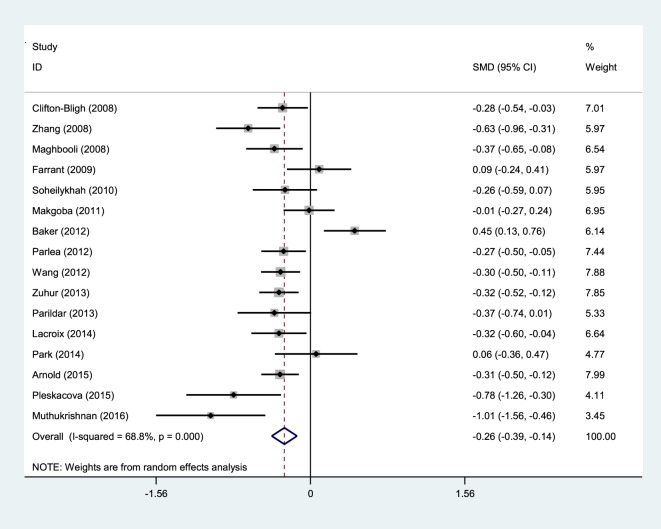
Relationship between serum 25(OH)D level and GDM.

Eighteen studies from 26 cases provided their findings as averages and means; 12 of these 18 studies reported a relationship between vitamin D deficiency and GDM. The current meta-analysis was performed to determine whether there is a meaningful difference between the average vitamin D levels in women with and those without gestational diabetes. According to the random effects model, the average standard equation difference was −2.26 (SMD) (CI at 95% = −0.39 to −0.14), which indicates that heterogeneity was significant (*I*^2^ = 68.8%, *p* < 0.001) (Figure 3).

These results showed that pregnant women with GDM have lower vitamin D values than the compared group.

A sensitive analysis was also performed in the current research. After each study was sequentially removed from the analysis, the relative risk of the overall composition did not change. Thus, it was concluded that no special study would affect the results.

**Figure 4:**
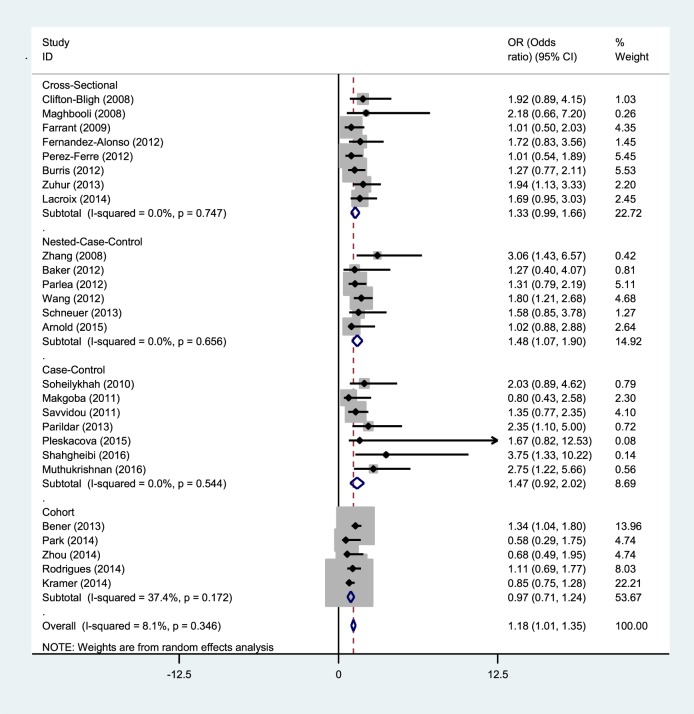
Relationship between serum 25(OH)D level and GDM based on study design.

A subgroup analysis of gestational diabetes was conducted in the current study to minimize heterogeneity among various studies. As can be seen in Figure 4, a study showed that vitamin D deficiency is correlated with an increased risk of gestational diabetes if the study did not use a cross-sectional and cohort design (Figure 4).

**Figure 5:**
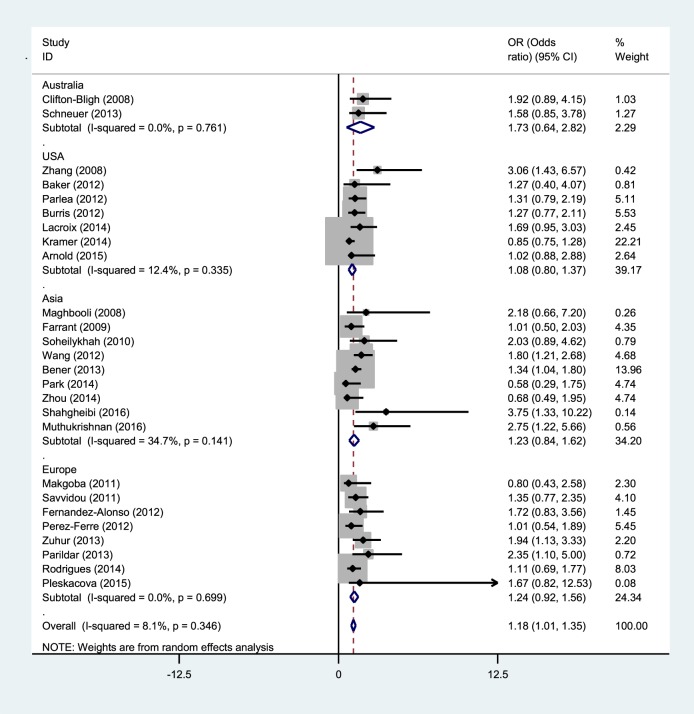
Relationship between serum 25(OH)D level and GDM based on region.

This relationship was also considered on the basis of region. Of the 26 studies, 2 were conducted in Australia (28, 43), 7 in North America (7, 16, 31, 33, 35, 36, 44), 9 in Asia (6, 15, 17, 23, 26, 32, 37, 38, 45), and 8 in Europe (24, 25, 27, 29, 30, 40, 46, 47). The results of the current meta-analysis showed that there is no significant relationship between the status of vitamin D and the risk of gestational diabetes based on geographical area (Figure 5).

### Publication Bias

**Figure 6:**
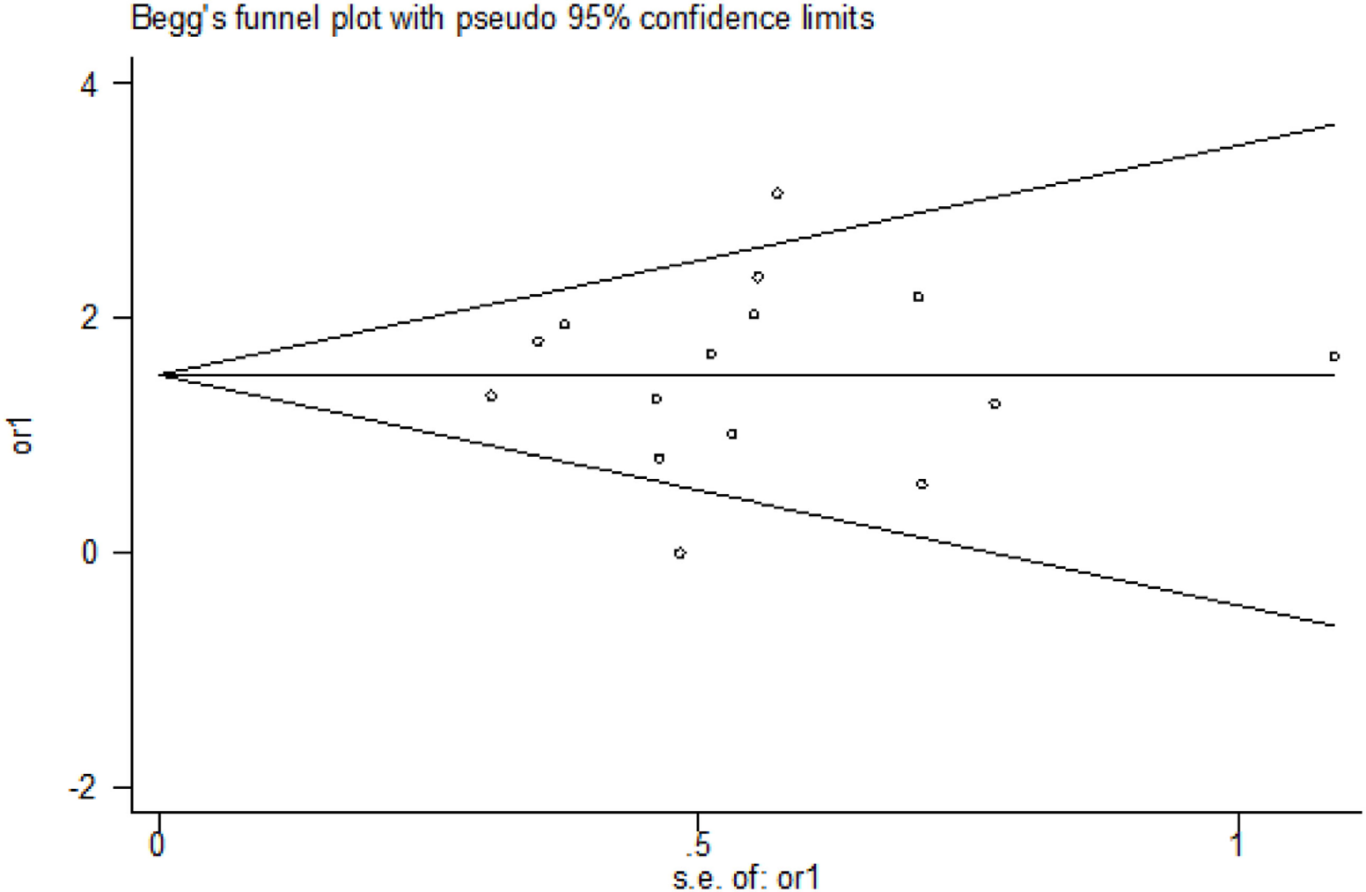
Funnel plot (from Begg–Mazumdar test) for publication bias.

**Figure 7:**
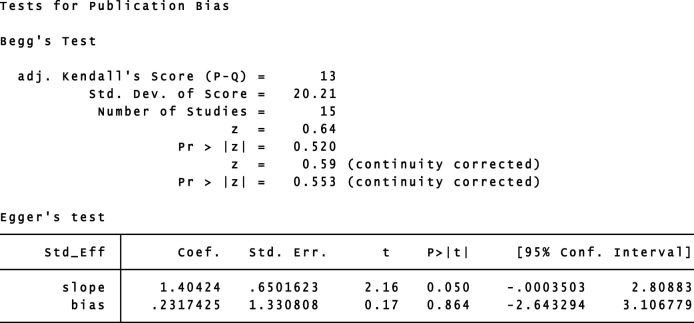
Begg–Mazumdar rank correlation and Egger regression tests for publication bias. OR, odds ratio.

The funnel plots presented in the Begg–Mazumdar test showed no signs of publication bias in the studies considered. Figure 6 shows the shape of funnel plots of tests related to vitamin D deficiency in patients with GDM. Egger and Begg tests showed no evidence of publication bias (*p* = 0.864 for Egger; *p* = 0.553 for Begg’s; Figure 7).

## Discussion

The current systematic study and quantitative meta-analysis found a relationship between the rate of vitamin D deficiency and gestational diabetes from 26 observational studies. The current results were consistent with those of previous meta-analyses. A 2012 meta-analysis reported from 7 observational studies that vitamin D deficiency in relation with GDM is meaningful and significant; it should be added, however, that according to an entrance study, this relationship remains unclear (18). A 2013 meta-analysis from 10 observational studies showed that there is a significant association between vitamin D deficiency and undesirable results in gestation and birth variables (19). In a 2015 meta-analysis from 20 observational studies, the relationship between vitamin D deficiency and an increased risk of GDM was confirmed (20). A 2016 subsequent meta-analysis reported a meaningful reverse relationship between the level of vitamin D and the risk of gestational diabetes (21).

We observed that vitamin D deficiency had a positive association with GDM in 7 of 26 studies and 19 of 26 studies did not support this conclusion; however, our results showed a mild positive association between vitamin D status with GDM. In previous meta-analyses also have such a case (19–21). It should be mentioned that in meta-analysis sample size and SD are very important in combining the results of studies, in extreme situation maybe more studies do not show significance association but few studies with big sample size show big effect size and significance results. This matter affected our and previous meta-analyses.

As previously mentioned, there have been further developments in the subject literature since the publication of these meta-analyses, and the previous meta-analyses did not include several important observational studies; the current meta-analysis is an attempt to fill this gap. The previous meta-analysis showed that vitamin D insufficiency was associated with increased gestational diabetes risk by 45% (21), but we added some studies to previous meta-analysis and concluded that vitamin D insufficiency increased the risk of gestational diabetes by 18%. The current findings suggest softening the conclusion in previous meta-analysis and show that vitamin D deficiency may increase the risk of gestational diabetes.

In the present study, we found that the serum level of 25(OH)D was significantly lower in women with gestational diabetes than in women with NGT. Most of the reviewed studies reported that pregnant women with GDM have lower vitamin D values than the compared group (7, 15, 17, 25, 33, 36, 37, 40, 43, 44). The previous meta-analyses also evaluated weighted mean differences between the average vitamin D levels in women with and those without gestational diabetes and their results were consistent with ours (18, 20). According to these findings, it can be said that low levels of 25(OH)D may be considered a risk factor during gestation.

It is important to note that the relationship between the mother’s vitamin D status and the risk of gestational diabetes can be affected by various factors. Some possible contributing factors are mother’s age and body mass index (BMI). Different studies have shown a correlation between gestational diabetes and a mother’s high age, high BMI, and reduced physical activity (41, 48). Obesity and high age in the mother are independent risk factors for both vitamin D deficiency and gestational diabetes; consequently, they can be considered as means of the relationship between vitamin D deficiency and gestational diabetes (18). More entrance studies in the current meta-analysis were regulated on the basis of mother’s age and BMI. However, the relationship of vitamin D status and gestational diabetes was statistically significant.

The consumption of pre-natal multivitamins or nutritional supplements is one of the factors that can affect the relationship between maternal vitamin D status and the risk of developing gestational diabetes. A systematic study and meta-analysis showed that daily vitamin D supplementation (800–1,000 IU per day) has protective effects on maternal health outcomes before and after childbirth and/or on the newborn (42). The researchers also showed that vitamin D supplementation during the gestational period has a positive effect on glycemic, sensitivity, and insulin resistance as well as metabolic characteristics (34, 39, 49).

Other contributing factors or possibly risky factors may be exposure to sunlight, which has an intensive relationship with geographical situation, season, and weather; skin color and the use of sunscreen; and even air pollution (50). Recent evidence has shown that inadequate vitamin D levels are completely common in pregnant women who have limited exposure to sunlight. Some of the sample people who may be less exposed to sunlight are people who live in a geographical area with cold weather, those who wear clothes that are protective against sunlight, and/or those who are from ethnic groups of darker-colored skin (51–53). First, vitamin D is produced in skin which is exposed to ultraviolet B rays that stimulate the production of serum levels of 25(OH)D depending on the season; higher levels are seen in spring and summer (18).

Other factors can also be related to the risk of gestational diabetes, including smoking, alcohol consumption, weight gain associated with pregnancy, and social and economic status. The contribution of these confounding factors to the risk of GDM can go some distance in explaining the conflicting results among different studies.

In the current study, subgroup analysis based on the research project of various studies in the meta-analysis showed that inadequate vitamin D is correlated with an increased risk of gestational diabetes if the studies did not use a cross sectional and cohort design. Since many of the studies reviewed in the present work had a case–control design, this may have led to overestimation of the effect size of the association. Similarly, previous meta-analyses confirmed the current results in this regard, showing that study design can be one explanation for heterogeneity among studies included in a meta-analysis (19–21).

Another significant point is that entrance studies had used different methods and standards to recognize gestational diabetes. On the other hand, serum level 25(OH)D was measured in different quarterly courses, the definitions of deficiency or inefficiency of 25(OH)D were different, and different techniques were used. Some studies suggest that the method used to quantify 25(OH)D levels and the diagnosis of gestational diabetes could be an important factor and influence the final results (54, 55).

The possible mechanisms related to the relationship between vitamin D and gestational diabetes are as follows: (1) The direct effect of vitamin D on the performance of β cells of the pancreas has a regulating effect on the rate of circulating glucose in the blood which is transmitted through the connection of 1,25(OH)_2_D_3_ to vitamin D receiver of β cells of the pancreas. Glucose stimulates the expression of insulin receptors and the 25-hydroxyvitamin D-1-α-hydroxylase enzyme and increases the insulin response for transfusion (56–58). (2) The effect on intracellular calcium regulation, which plays a major and important role in insulin-mediated intracellular processes of insulin-receptor tissue (59). (3) Effects on systemic inflammation along with insulin resistance in patients with diabetes mellitus (60).

### Limitation

This meta-analysis had several limitations. First, there were some differences among studies regarding the definition of the limits for vitamin D deficiency and the criteria for a diagnosis of GDM, which may be affected by accumulation. Second, the impact of some of the most important factors that may affect the relationship between vitamin D deficiency and gestational diabetes, including weight gain in pregnancy, skin color, and socioeconomic status, could not be evaluated due to inadequate data and information in some studies. Data on exposure to sunlight and vitamin D intake were not available. Third, the most adjusted OR was used in this meta-analysis. However, modulated models varied in different studies. Also, in some studies, the potential confounding factors could not be adjusted; so, the findings cannot be collected by adjusting the confounding factors. In addition, in meta-analysis sample size and SD are very important in combining the results of studies; this subject affected our study. Furthermore, the possible effects of publication bias inherent in any meta-analysis cannot be ruled out.

## Conclusion

The present meta-analysis shows that women with GDM had significantly less serum vitamin D than women who have NGT. The current finding shows that low levels of 25(OH)D may be considered a risk factor during gestation. Also, the results showed that there is a mild positive association between maternal vitamin D deficiency and GDM and suggested softening the reported conclusion in previous meta-analysis. In conclusion, our results show that vitamin D deficiency may increase the risk of gestational diabetes. Based on different research projects and the heterogeneity among various studies, it is not logical to make a definitive conclusion. More clinical trials and studies are needed to determine the effects of vitamin D supplements on the prevention of gestational diabetes. Based on these findings, healthcare providers should encourage pregnant women to follow the guidelines for the daily intake of vitamin D.

## Ethics Statement

This study has a code of ethics by the Ethics Committee of Ilam University of Medical Sciences (ir.medilam.rec.1395.102).

## Author Contributions

MA and SM designed the study and participated in data collection; KS, AM, and ES performed the meta-analysis; AM, SFM, ES, and SM participated in drafting the paper; KS and ES critically revised the paper; all authors provided data analysis, read, and approved the manuscript.

## Conflict of Interest Statement

The authors declare that the research was conducted in the absence of any commercial or financial relationships that could be construed as a potential conflict of interest.

## References

[1] World Health Organization. In Diagnostic Criteria and Classification of Hyperglycemia First Detected in Pregnancy. WHO/NMH/MND/13. 2 ed Geneva: World Health Organization (2013). 24199271

[2] Hao M Lin L. Fasting plasma glucose and body mass index during the first trimester of pregnancy as predictors of gestational diabetes mellitus in a Chinese population. Endocr J (2017) 64(5):561–9. 10.1507/endocrj.EJ16-0359 28420856

[3] Moyer VA U.S. Preventive Services Task Force. Screening for gestational diabetes mellitus: U.S. Preventive Services Task Force recommendation statement. Ann Intern Med (2014) 160:414–20. 10.7326/M13-2905 24424622

[4] Metzger BE Lowe LP Dyer AR Trimble ER Chaovarindr U Coustan DR Hyperglycemia and adverse pregnancy outcomes. N Engl J Med (2008) 358:1991–2002. 10.1056/NEJMoa0707943 18463375

[5] Yogev Y Chen R Hod M Coustan DR Oats JJN McIntyre HD Hyperglycemia and adverse pregnancy outcome (HAPO) study: preeclampsia. Am J Obstet Gynecol (2010) 202:255.e1–7. 10.1016/j.ajog.2010.01.024 20207245 PMC2836485

[6] Shahgheibi S Farhadifar F Pouya B. The effect of vitamin D supplementation on gestational diabetes in high-risk women: results from a randomized placebo-controlled trial. J Res Med Sci (2016) 21:2. 10.4103/1735-1995.175148 27904548 PMC5122001

[7] Lacroix M Battista MC Doyon M Houde G Menard J Ardilouze JL Lower vitamin D levels at first trimester are associated with higher risk of developing gestational diabetes mellitus. Acta Diabetol (2014) 51:609–16. 10.1007/s00592-014-0564-4 24526261

[8] Bikle D. Nonclassic actions of vitamin D. J Clin Endocrinol Metab (2009) 94:26–34. 10.1210/jc.2008-1454 18854395 PMC2630868

[9] Yap C Cheung NW Gunton JE Athayde N Munns CF Duke A Vitamin D supplementation and the effects on glucose metabolism during pregnancy: a randomized controlled trial. Diabetes Care (2014) 37:1837–44. 10.2337/dc14-0155 24760259

[10] Gernand AD Simhan HN Caritis S Bodnar LM. Maternal vitamin D status and small-for-gestational-age offspring in women at high risk for preeclampsia. Obstet Gynecol (2014) 123:40–8. 10.1097/AOG.0000000000000049 24463662 PMC3914014

[11] Grant CC Stewart AW Scragg R Milne T Rowden J Ekeroma A Vitamin D during pregnancy and infancy and infant serum 25-hydroxyvitamin D concentration. Pediatrics (2014) 133:e143–53. 10.1542/peds.2013-2602 24344104

[12] Robinson CJ Alanis MC Wagner CL Hollis BW Johnson DD. Plasma 25-hydroxyvitamin D levels in early-onset severe preeclampsia. Am J Obstet Gynecol (2010) 203:.e1–6. 10.1016/j.ajog.2010.06.036 20692641 PMC3192365

[13] Merewood A Mehta SD Chen TC Bauchner H Holick MF. Association between vitamin D deficiency and primary cesarean section. J Clin Endocrinol Metab (2009) 94:940–5. 10.1210/jc.2008-1217 19106272 PMC2681281

[14] Jain M Kapry S Jain S Singh SK Singh TB Maternal vitamin D deficiency: a risk factor for gestational diabetes mellitus in North India. Gynecol Obstet (2015) 5:264 10.4172/2161-0932.1000264

[15] Maghbooli Z Hossein-Nezhad A Karimi F Shafaei AR Larijani B. Correlation between vitamin D3 deficiency and insulin resistance in pregnancy. Diabetes Metab Res Rev (2008) 24:27–32. 10.1002/dmrr.737 17607661

[16] Kramer CK Swaminathan B Hanley AJ Connelly PW Sermer M Zinman B Vitamin D and parathyroid hormone status in pregnancy: effect on insulin sensitivity, beta cell function, and gestational diabetes mellitus. J Clin Endocrinol Metab (2014) 99:4506–13. 10.1210/jc.2014-2341 25202819

[17] Muthukrishnan J Dhruv G Vitamin D status and gestational diabetes mellitus. Indian J Endocrinol Metab (2015) 19:616–9. 10.4103/2230-8210.163175 26425469 PMC4566340

[18] Poel YH Hummel P Lips P Stam F van der Ploeg T Simsek S. Vitamin D and gestational diabetes: a systematic review and meta-analysis. Eur J Intern Med (2012) 23:465–9. 10.1016/j.ejim.2012.01.007 22726378

[19] Aghajafari F Nagulesapillai TH Ronksley PE Tough SC O’Beirne M Rabi DM. Association between maternal serum 25-hydroxyvitamin D level and pregnancy and neonatal outcomes: systematic review and meta-analysis of observational studies. BMJ (2013) 346:f1169. 10.1136/bmj.f1169 23533188

[20] Zhang MX Pan GT Guo JF Li BY Qin LQ Zhang ZL. Vitamin D deficiency increases the risk of gestational diabetes mellitus: a meta-analysis of observational studies. Nutrients (2015) 7:8366–75. 10.3390/nu7105398 26437429 PMC4632418

[21] Lu M Xu Y Lv L Zhang M. Association between vitamin D status and the risk of gestational diabetes mellitus: a meta-analysis. Arch Gynecol Obstet (2016) 293:959–66. 10.1007/s00404-016-4010-4 26825733

[22] Moher D Liberati A Tetzlaff J Altman DG PRISMA Group Preferred reporting items for systematic reviews and metaanalyses: the PRISMA statement. PLoS Med (2009) 6:e1000097 10.1371/journal.pmed.1000097 19621072 PMC2707599

[23] Soheilykhah S Mojibian M Moghadam MJ Shojaoddiny-Ardekani A. The effect of different doses of vitamin D supplementation on insulin resistance during pregnancy. Gynecol Endocrinol (2013) 29:396–9. 10.3109/09513590.2012.752456 23350644

[24] Savvidou MD Akolekar R Samaha RB Masconi AP Nicolaides KH. Maternal serum 25-hydroxyvitamin D levels at 11(+0) -13(+6) weeks in pregnant women with diabetes mellitus and in those with macrosomic neonates. BJOG (2011) 118:951–5. 10.1111/j.1471-0528.2011.02982.x 21658195

[25] Zuhur SS Erol RS Kuzu I Altuntas Y. The relationship between low maternal serum 25-hydroxyvitamin D levels and gestational diabetes mellitus according to the severity of 25-hydroxyvitamin D deficiency. Clinics (2013) 68:658–64. 10.6061/clinics/2013(05)13 23778416 PMC3654301

[26] Bener A Al-Hamaq AO Saleh NM. Association between vitamin D insufficiency and adverse pregnancy outcome: global comparisons. Int J Womens Health (2013) 5:523–31. 10.2147/IJWH.S51403 24043954 PMC3772690

[27] Parildar H Dogruk Unal A Aksan Desteli G Cigerli O Guvener Demirag N. Frequency of Vitamin D deficiency in pregnant diabetics at Baskent University Hospital, Istanbul. Pak J Med Sci (2013) 29:15–20. 10.12669/pjms.291.2896 24353500 PMC3809191

[28] Schneuer FJ Roberts CL Guilbert C Simpson JM Algert CS Khambalia AZ Effects of maternal serum 25-hydroxyvitamin D concentrations in the first trimester on subsequent pregnancy outcomes in an Australian population. Am J Clin Nutr (2014) 99:287–95. 10.3945/ajcn.113.065672 24257720

[29] Makgoba M Nelson SM Savvidou M Messow CM Nicolaides K Sattar N. First-trimester circulating 25-hydroxyvitamin D levels and development of gestational diabetes mellitus. Diabetes Care (2011) 34:1091–3. 10.2337/dc10-2264 21454797 PMC3114479

[30] Perez-Ferre N Torrejon MJ Fuentes M Fernandez MD Ramos A Bordiu E Association of low serum 25-hydroxyvitamin D levels in pregnancy with glucose homeostasis and obstetric and newborn outcomes. Endocr Pract (2012) 18:676–84. 10.4158/EP12025.OR 22548949

[31] Burris HH Rifas-Shiman SL Kleinman K Litonjua AA Huh SY Rich-Edwards JW Vitamin D deficiency in pregnancy and gestational diabetes mellitus. Am J Obstet Gynecol (2012) 207:.e1–8. 10.1016/j.ajog.2012.05.022 PMC3432741 22717271

[32] Park S Yoon HK Ryu HM Han YJ Lee SW Park BK Maternal vitamin D deficiency in early pregnancy is not associated with gestational diabetes mellitus development or pregnancy outcomes in Korean pregnant women in a prospective study. J Nutr Sci Vitaminol (Tokyo) (2014) 60:269–75. 10.3177/jnsv.60.269 25297616

[33] Arnold DL Enquobahrie DA Qiu C Huang J Grote N VanderStoep A Early pregnancy maternal vitamin D concentrations and risk of gestational diabetes mellitus. Paediatr Perinat Epdemiol (2015) 29:200–10. 10.1111/ppe.12182 PMC4400239 25808081

[34] Asemi Z Hashemi T Karamali M Samimi M Esmaillzadeh A. Effects of vitamin D supplementation on glucose metabolism, lipid concentrations, inflammation, and oxidative stress in gestational diabetes: a double-blind randomized controlled clinical trial. Am J Clin Nutr (2013) 98:1425–32. 10.3945/ajcn.113.072785 24132976

[35] Baker AM Haeri S Camargo CA Jr Stuebe AM Boggess KA First-trimester maternal vitamin D status and risk for gestational diabetes (GDM) a nested case–control study. Diabetes Metab Res Rev (2012) 28:164–8. 10.1002/dmrr.1282 21818838 PMC4381548

[36] Parlea L Bromberg IL Feig DS Vieth R Merman E Lipscombe LL. Association between serum 25-hydroxyvitamin D in early pregnancy and risk of gestational diabetes mellitus. Diabet Med (2012) 29:e25–32. 10.1111/j.1464-5491.2011.03550.x 22150870

[37] Wang O Nie M Hu YY Zhang K Li W Ping F Association between vitamin D insufficiency and the risk for gestational diabetes mellitus in pregnant Chinese women. Biomed Environ Sci (2012) 25:399–406. 10.3967/0895-3988.2012.04.004 23026519

[38] Zhou J Su L Liu M Liu Y Cao X Wang Z Associations between 25-hydroxyvitamin D levels and pregnancy outcomes: a prospective observational study in southern China. Eur J Clin Nutr (2014) 68:925–30. 10.1038/ejcn.2014.99 24865483

[39] Asemi Z Samimi M Tabassi Z Shakeri H Esmaillzadeh A. Vitamin D supplementation affects serum high-sensitivity C-reactive protein, insulin resistance, and biomarkers of oxidative stress in pregnant women. J Nutr (2013) 143:1432–8. 10.3945/jn.113.177550 23884390

[40] Pleskacova A Bartakova V Pacal L Kuricova K Belobradkova J Tomandl J Vitamin D status in women with gestational diabetes mellitus during pregnancy and postpartum. Biomed Res Int (2015) 2015:260624. 10.1155/2015/260624 26000285 PMC4427001

[41] Tobias DK Zhang C van Dam RM Bowers K Hu FB. Physical activity before and during pregnancy and risk of gestational diabetes mellitus: a meta-analysis. Diabetes Care (2011) 34:223–9. 10.2337/dc10-1368 20876206 PMC3005457

[42] Thorne-Lyman A Fawzi WW. Vitamin D during pregnancy and maternal, neonatal and infant health outcomes: a systematic review and meta-analysis. Paediatr Perinat Epidemiol (2012) 26:75–90. 10.1111/j.1365-3016.2012.01283.x 22742603 PMC3843348

[43] Clifton-Bligh RJ McElduff P McElduff A. Maternal vitamin D deficiency, ethnicity and gestational diabetes. Diabet Med (2008) 25:678–84. 10.1111/j.1464-5491.2008.02422.x 18544105

[44] Zhang C Qiu C Hu FB David RM van Dam RM Bralley A Maternal plasma25-hydroxyvitamin D concentrations and the risk for gestational diabetes mellitus. PLoS One (2008) 3:e3753. 10.1371/journal.pone.0003753 19015731 PMC2582131

[45] Farrant HJ Krishnaveni GV Hill JC Boucher BJ Fisher DJ Noonan K Vitamin D insufficiency is common in Indian mothers but is not associated with gestational diabetes or variation in newborn size. Eur J Clin Nutr (2009) 63:646–52. 10.1038/ejcn.2008.14 18285809 PMC2678985

[46] Fernandez-Alonso AM Dionis-Sanchez EC Chedraui P Gonzalez-Salmeron MD Perez-Lopez FR First-trimester maternal serum 25-hydroxyvitamin D3 status and pregnancy outcome. Int J Gynaecol Obstet (2012) 116:6–9. 10.1016/j.ijgo.2011.07.029 21959069

[47] Rodriguez A Garcia-Esteban R Basterretxea M Lertxundi A Rodriguez-Bernal C Iniguez C Associations of maternal circulating 25-hydroxyvitamin D3 concentration with pregnancy and birth outcomes. BJOG (2015) 122:1695–704. 10.1111/1471-0528.13074 25208685

[48] Prentice A Goldberg GR Schoenmakers I. Vitamin D across the lifecycle: physiology and biomarkers. Am J Clin Nutr (2008) 88:500S–6S. 18689390 10.1093/ajcn/88.2.500S

[49] Soheilykhah S Mojibian M Rashidi M Rahimi-Saghand S Jafari F. Maternal vitamin D status in gestational diabetes mellitus. Nutr Clin Pract (2010) 25:524–7. 10.1177/0884533610379851 20962313

[50] Wacker M Holick MF Sunlight and vitamin D: a global perspective for health. Dermatoendocrinol (2013) 5:51–108. 10.4161/derm.24494 24494042 PMC3897598

[51] Hollis BW Wagner CL. Assessment of dietary vitamin D requirements during pregnancy and lactation. Am J Clin Nutr (2004) 79:717–26. 15113709 10.1093/ajcn/79.5.717

[52] Lee JM Smith JR Philipp BL Chen TC Mathieu J Holick MF. Vitamin D deficiency in a healthy group of mothers and newborn infants. Clin Pediatr (Phila) (2007) 46:42–4. 10.1177/0009922806289311 17164508

[53] Bodnar LM Simhan HN Powers RW Frank MP Cooperstein E Roberts JM. High prevalence of vitamin D insufficiency in black and white pregnant women residing in the northern United States and their neonates. J Nutr (2007) 137:447–52. 17237325 10.1093/jn/137.2.447 PMC4288960

[54] Shirazian N Mahboubi M Emdadi R Yousefi-Nooraie R Fazel-Sarjuei Z Sedighpour N. Comparison of different diagnostic criteria for gestational diabetes mellitus based on the 75-g oral glucose tolerance test: a cohort study. Endocr Pract (2008) 14:312–7. 10.4158/EP.14.3.312 18463038

[55] Agarwal MM Dhatt GS Punnose J Koster G. Gestational diabetes: dilemma caused by multiple international diagnostic criteria. Diabet Med (2005) 22:1731–6. 10.1111/j.1464-5491.2005.01706.x 16401320

[56] Chiu KC Chu A Go VL Saad MF. Hypovitaminosis D is associated with insulin resistance and beta cell dysfunction. Am J Clin Nutr (2004) 79:820–5. 15113720 10.1093/ajcn/79.5.820

[57] Norman AW Frankel JB Heldt AM Grodsky GM. Vitamin D deficiency inhibits pancreatic secretion of insulin. Science (1980) 209:823–5. 10.1126/science.6250216 6250216

[58] Vaidya A Williams JS Vitamin D and insulin sensitivity: can gene association and pharmacogenetic studies of the vitamin d receptor provide clarity? Metab Clin Exp (2012) 61:759–61. 10.1016/j.metabol.2011.12.009 22304841 PMC4277866

[59] Draznin B Sussman KE Eckel RH Kao M Yost T Sherman NA. Possible role of cytosolic free calcium concentrations in mediating insulin resistance of obesity and hyperinsulinemia. J Clin Invest (1988) 82:1848–52. 10.1172/JCI113801 3143744 PMC442763

[60] Alvarez JA Ashraf A. Role of vitamin D in insulin secretion and insulin sensitivity for glucose homeostasis. Int J Endocrinol (2010) 2010:351385. 10.1155/2010/351385 20011094 PMC2778451

